# Angiotensin II Type 1 Receptor Blockers Reduce Urinary Angiotensinogen Excretion and the Levels of Urinary Markers of Oxidative Stress and Inflammation in Patients with Type 2 Diabetic Nephropathy

**DOI:** 10.4137/bmi.s2733

**Published:** 2009-06-23

**Authors:** Susumu Ogawa, Hiroyuki Kobori, Naro Ohashi, Maki Urushihara, Akira Nishiyama, Takefumi Mori, Tsuneo Ishizuka, Kazuhiro Nako, Sadayoshi Ito

**Affiliations:** 1 Division of Nephrology, Endocrinology, and Vascular Medicine, Department of Medicine, Tohoku University hospital, Sendai, Japan; 2 Department of Physiology, and Hypertension and Renal Center of Excellence, Tulane University Health Sciences Center, New Orleans, LA; 3 Department of Pharmacology, Kagawa University Medical School, Kagawa, Japan., Email: ogawa-s@mail.tains.tohoku.ac.jp

**Keywords:** angiotensin II type 1, receptor blockers, diabetic nephropathy

## Abstract

**Objective:**

To demonstrate that the administration of an angiotensin (Ang) II type 1 receptor (AT1R) blocker (ARB) inhibits the vicious cycle of high glucose (HG)-reactive oxygen species (ROS)-angiotensinogen (AGT)-Ang II-AT1R-ROS by suppressing ROSs and inflammation, thus ameliorating diabetic nephropathy (DN).

**Research Design and Methods:**

Thirteen hypertensive DN patients were administered ARBs, and the following parameters were evaluated before and 16 weeks after the treatment: urinary AGT (UAGT), albumin (albumin-creatinine ratio: ACR), 8-hydroxyde-oxyguanosine (8-OHdG), 8-epi-prostaglandin F2α(8-epi-PGF2α), monocyte chemoattractant protein (MCP)-1, interleukin (IL)-6, and IL-10.

**Results:**

ARB treatment reduced the blood pressure and urinary levels of AGT, ACR, 8-OHdG, 8-epi-PGF2α, MCP-1, and IL-6 but increased the urinary levels of IL-10. The reduction rate of UAGT correlated with the reduction rate of blood pressure; the reduction rates of the urinary ACR, 8-OHdG, 8-epi-PGF2α, MCP-1, and IL-6 levels; and the increase rate of the urinary IL-10 levels. Moreover, subjects who had high UAGT values at baseline exhibited higher reduction rates of urinary albumin excretion.

**Conclusions:**

ARB-induced blockade of the abovementioned vicious cycle contributes to the renoprotective effects of ARBs in DN. The urinary levels of AGT could represent a predictive factor for reduced ACR in patients receiving ARB treatment.

## Introduction

High glucose (HG) levels lead to increased angiotensin (Ang) II generation in the kidney. Reactive oxygen species (ROSs) mediate a process that increases the levels of angiotensinogen (AGT), leading to increased Ang II generation.^1^–^3^ HG induces AGT generation via the ROS–AGT pathway.^4^–^6^ AGT generation mediated by HG-induced ROSs, accompanied by increased local production of Ang II, causes many pathophysiological changes associated with diabetic nephropathy (DN).^7^–^9^ Ang II induces a further increase in the intracellular ROS levels via the activation of the Ang II type 1 receptor (AT1R).^10^–^11^ Increased ROS levels, whatever they may be attributed to, cause inflammation and renal damage. Monocyte chemoattractant protein (MCP)-1 and interleukin (IL)-6 are assumed to be the key inflammatory factors involved in these effects. The renoprotective action of an AT1R blocker (ARB) is strongly related to its ROS-lowering effects.^10^ In DN, the generation of ROS, AGT, and Ang II seems to increase remarkably when the vicious cycle of HG-ROS-AGT-Ang II-AT1R-ROS is activated.^1^–^9^

An ARB is expected to block this vicious cycle and thus decrease the urinary levels of inflammatory markers, ROS markers, albumin (measured as the albumin-creatinine ratio [ACR]), and AGT. Furthermore, all such effects of an ARB are expected to be closely correlated. However, these effects have not yet been clinically investigated. The present study was designed to confirm that AT1R blockade reduces the urinary AGT (UAGT) levels and to determine whether changes in these levels are closely related to changes in the ACR and in the levels of urinary ROSs and inflammatory markers. We therefore determined the ACR and the levels of UAGT, inflammatory markers, and ROSs before and after the administration of ARB to DN patients.

## Research Design and Methods

The subjects enrolled in the present study were outpatients with hypertensive type 2 DN at our hospital. The enrollment criteria were as follows: mild to moderate hypertension (office systolic blood pressure (SBP) = 130–199 and/or diastolic blood pressure (DBP) = 70–110 mmHg), no use of antihypertensive agents such as RAS inhibitors, HbA_1c_ levels < 8%, ACR > 30 μg/mg Cre, and absence of hematuria. The present study was conducted after we obtained informed consent from all the subjects, and the study protocol was approved by the ethics committees of Tohoku University Hospital. Although 20 patients were initially enrolled, 7 were subsequently excluded for various reasons. Thus, the study finally involved 13 patients (7 men and 6 women) who had had diabetes for 13.4 ± 3.7 years. The subjects were administered ARBs (olmesartan in the case of 7 patients and valsartan in the case of 6). The following parameters were measured before and 16 weeks after the treatment: UAGT; oxidative stress markers such as 8-epi-prostaglandin F2α (8-epi-PGF2α ) and 8-hydroxydeoxyguanosine (OHdG); the inflammatory markers MCP-1, IL-6, and IL-10; and the ACR.^10^ The UAGT levels were determined using a newly developed ELISA.^12^–^14^ The ACR and the levels of UAGT, 8-epi-PGF2α , 8-OHdG, MCP-1, IL-6, and IL-10 were expressed in terms of the median (range), because these factors did not exhibit normal distribution. The difference between the values determined before and after the treatment was analyzed using the Wilcoxon signed-rank test. All the other data were expressed as the mean ± standard error of the mean (SEM) and were statistically analyzed using the paired Student *t* test. Correlations were determined using the Spearman rank correlation test. p < 0.05 was considered significant.

## Results

**Figure 1:**
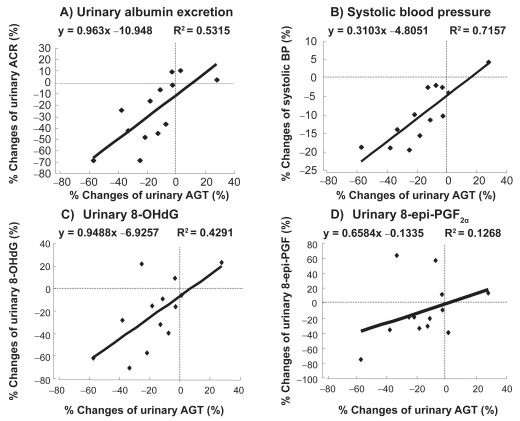
Correlation between the reduction rate of the urinary angiotensinogen (UAGT) levels and that of urinary albumin excretion (ACr): **A**) reduction rate of systolic blood pressure (SBp): **B**) reduction rates of the urinary 8-hydroxydeoxyguanosine (8-ohdG) and urinary 8-epi-prostaglandin F2α (8-epi-pGF2α) levels.

**Figure 2:**
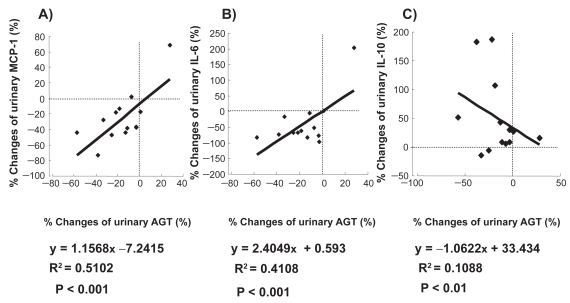
Correlation between the reduction rate of the urinary angiotensinogen (UAGT) levels and those of the monocyte chemoattractant protein (MCp)-1 **A**) interleukin (IL)-6 **B**), and IL-10 (**C**) levels.

**Figure 3:**
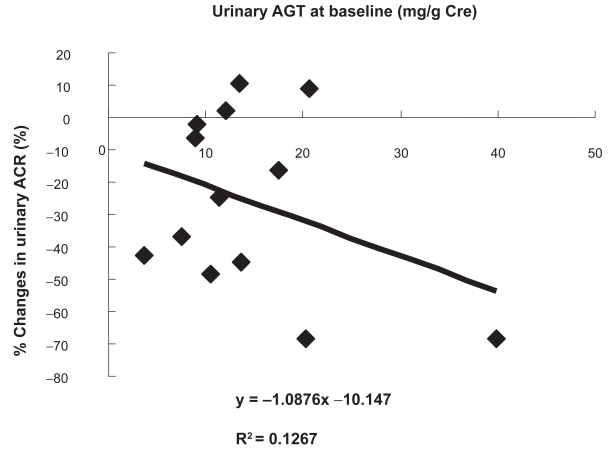
Correlation between the baseline values of urinary angiotensinogen (UAGT) and the reduction rate of urinary albumin excretion (ACr).

The changes in the clinical parameters evaluated before and after ARB administration were as follows: body mass index (kg/m^2^), from 22.4 ± 0.7 to 22.5 ± 0.6 (not significant, p = 0.8994); HbA_1c_ (%), from 6.6 ± 0.3 to 6.6 ± 0.2 (not significant, p = 0.9804); SBP (mmHg), from 161.6 ± 2.8 to 145.7 ± 3.1 (− 9.7% ± 2.2%, p < 0.01); DBP (mmHg), from 80.8 ± 1.8 to 78.6 ± 1.9 (− 2.2% ± 2.3%, p < 0.01); serum creatinine (mg/dl), from 1.1 ± 0.1 to 1.3 ± 0.1 (p < 0.05); and K^+^ (mEq/l), from 5.0 ± 0.2 to 5.3 ± 0.2 (p < 0.05). The ACR and the urinary levels of AGT, 8-epi-PGF2α , 8-OHdG, MCP-1, and IL-6 were significantly reduced (Table 1). However, the plasma levels of these markers remained unchanged (data not shown). The percent changes in the parameters evaluated before and after ARB administration are shown in Table 1. The reduction rate of the UAGT levels correlated with the reduction rates of the ACR (y = 0.963x –10.948, r = 0.7290, p < 0.001; Fig. 1A) and the SBP (y = 0.3103x −4.8051, r = 0.8460, p < 0.001, Fig. 1B); the reduction rates of the 8-OHdG (y = 0.9488x −6.9257, r = 0.6551, p < 0.01; Fig. 1C), 8-epi-PGF2α (y = 0.6584x −0.1335, r = 0.3561, p < 0.01; Fig. 1D), MCP-1 (y = 1.1568x −7.2415, r = 0.7143, p < 0.001; Fig. 2A), and IL-6 (y = 2.4049x +0.593, r = 0.6409, p < 0.001; Fig. 2B) levels; and the increase rate of the IL-10 levels (y = −1.0622x + 33.434, r= −0.3299, p < 0.01; Fig. 2–C). Subjects who had high UAGT levels at baseline exhibited a high ACR reduction rate (y = −1.0876x −10.147, r = −0.3599, p < 0.05; Fig. 3).

## Conclusions

The results of the present study revealed that a vicious cycle of HG-ROS-AGT-Ang II-AT1R-ROS may operate in DN. The continuous increase in the ROS levels contributes to the progression of DN. ARB treatment is believed to suppress ROS production by blocking the Ang II-AT1R-ROS pathway, thus reducing inflammation and suppressing AGT generation.^1^–^11^

We previously reported that a reduction in the urinary ROS levels is important for ARBs to exert their ACR-lowering effects, and that even with an equal reduction in the blood pressure; ARBs reduce the ROS levels more effectively than other antihypertensive agents.^10^ Thus, the action of ARBs in reducing the ROS levels is independent of a decrease in the blood pressure. Accordingly, it seems that the ROS reduction observed in this study may be attributed to the blockade of the Ang II-ROS pathway rather than to decrease in blood pressure. The findings of this study reveal that changes in the UAGT levels largely influence the ARB-induced changes in the ROS levels and ACR. The UAGT levels are reported to be elevated in hypertensive patients and are correlated with the ACR; further, ARB treatment reduces the UAGT levels.^12^ Changes in the UAGT levels are correlated with changes in not only the ACR but also the blood pressure; this suggests that AGT in the blood might be filtered in the glomeruli and excreted in urine. Thus, a reduction in the UAGT levels may merely reflect a decrease in the intraglomerular pressure rather than the suppression of AGT production. However, the ARB-induced reduction in the ACR is probably caused by blockade of the ROS-AGT-Ang II-AT1R-ROS cycle. This is because 1) the reduction in the UAGT levels correlated not only with the changes in the ACR and blood pressure but also with the changes in the levels of ROS and inflammatory markers, and 2) AGT is most probably produced in renal cells and released in urine.^1^–^8^ Another possible reason for reduced UAGT excretion may be that ARB increases the renal tubular reabsorption of AGT. Unfortunately, we were unable to investigate this possibility in the present study.

We have previously reported that the efficacy of ARBs increases with urinary oxidative stress.^10^ Moreover, we confirmed that the greater the UAGT excretion, the greater is the ACR suppression induced by ARB administration. The fact that the subjects in the present study exhibited increased UAGT and ROS levels indicates that the ROS-AGT-Ang II-AT1R-ROS cycle was strongly activated in these subjects. The stronger the activation of this cycle, the more prominent may be the effects of AT1R blockade. Activation of the AGT-Ang II-ROS pathway increases the intraglomerular pressure and the ACR. Therefore, the RAS-suppressive effects of ARB are believed to increase with UAGT excretion. Considering these implications of ROSs and AGT, we expect that the efficacy of ARBs increases with the UAGT levels.^10^ If this is true, UAGT could serve as a predictive factor for the renoprotective effects of ARBs.

The sample size of this study was too small for us to arrive at definitive conclusions; further investigation with a larger sample size is necessary. Furthermore, this study was not a randomized control trial (RCT) comparing ARBs with controls. In addition, since we did not perform examinations like a renal biopsy, we cannot be certain that the DN did in fact improve in our patients. Further clinical research is necessary for clarifying these issues. This research is a preliminary investigation and definitely needs to be followed up with a large-scale RCT. A large-scale RCT of this type, designated as the ORION-ANGEL (Olmesartan Reduces Inflammation and Oxidative stress in Nephropathy and suppresses ANGiotensinogen ELevation) Study (UMIN000001618), is currently underway.

## Figures and Tables

**Table 1:** Urinary parameters evaluated before and 16 weeks after the administration of ARBs.

Parameters	Unit	Before	After	p	% Change
AGT	mg/g Cre	12.0 (3.7–39.9)	8.9 (2.5–29.8)	<0.05	−15.6 ± 5.8
albumin	mg/g Cre	2026 (894–3428)	1232 (548–3783)	<0.01	−25.9 ± 7.7
8-epi-PGF2α	ng/g Cre	247 (124–802)	207 (113–454)	<0.05	−10.4 ± 10.7
8-oHdG	μg/g Cre	8.7 (4.3–17.5)	5.4 (4.4–14.8)	<0.01	−21.7 ± 8.4
MCP-1	ng/g Cre	575 (51–2170)	652 (37–2620)	<0.05	−25.4 ± 9.4
IL-6	ng/g Cre	9.7 (0.2–36.5)	0.8 (0.2–38.5)	<0.01	−36.3 ± 21.8
IL-10	ng/g Cre	0.8 (0.3–2.8)	0.9 (0.3–6.3)	<0.01	−50.0 ± 18.7

**Notes:** The parameters are expressed in term of the median (range). The percent change in the values determined after ARB administration as compared to those before administration is expressed as the mean ± SEM.

**Abbreviations:** AGT, angiotensinogen; 8-epi-PGF2α, 8-epi-prostaglandin F2α; 8-OHdG, 8-hydroxydeoxyguanosine; MCP, monocyte chemoattractant protein; Cre, urinary creatinine; IL, interleukin.
